# Robust multi-group gene set analysis with few replicates

**DOI:** 10.1186/s12859-016-1403-0

**Published:** 2016-12-09

**Authors:** Pashupati P. Mishra, Alan Medlar, Liisa Holm, Petri Törönen

**Affiliations:** 1 Institute of Biotechnology, University of Helsinki, P.O. Box 56, Viikinkaari 5, Helsinki, 00014 Finland; 2 Department of Biosciences, University of Helsinki, Viikinkaari 1, Helsinki, 00014 Finland

**Keywords:** Gene set analysis, Gene expression, Permutation

## Abstract

**Background:**

Competitive gene set analysis is a standard exploratory tool for gene expression data. Permutation-based competitive gene set analysis methods are preferable to parametric ones because the latter make strong statistical assumptions which are not always met. For permutation-based methods, we permute samples, as opposed to genes, as doing so preserves the inter-gene correlation structure. Unfortunately, up until now, sample permutation-based methods have required a minimum of six replicates per sample group.

**Results:**

We propose a new permutation-based competitive gene set analysis method for multi-group gene expression data with as few as three replicates per group. The method is based on advanced sample permutation technique that utilizes all groups within a data set for pairwise comparisons. We present a comprehensive evaluation of different permutation techniques, using multiple data sets and contrast the performance of our method, mGSZm, with other state of the art methods. We show that mGSZm is robust, and that, despite only using less than six replicates, we are able to consistently identify a high proportion of the top ranked gene sets from the analysis of a substantially larger data set. Further, we highlight other methods where performance is highly variable and appears dependent on the underlying data set being analyzed.

**Conclusions:**

Our results demonstrate that robust gene set analysis of multi-group gene expression data is permissible with as few as three replicates. In doing so, we have extended the applicability of such approaches to resource constrained experiments where additional data generation is prohibitively difficult or expensive. An R package implementing the proposed method and supplementary materials are available from the website http://ekhidna.biocenter.helsinki.fi/downloads/pashupati/mGSZm.html.

**Electronic supplementary material:**

The online version of this article (doi:10.1186/s12859-016-1403-0) contains supplementary material, which is available to authorized users.

## Background

Gene set analysis is an increasingly popular methodological approach for the analysis of molecular profiles such as gene expression [1, 2], metabolomics [3, 4] and genome-wide association data [5, 6]. In gene set analysis, statistical tests are performed on groups of genes that share characteristics defined by prior biological knowledge, for example, biological function [7] or regulatory pathway involvement [8]. A typical gene set analysis method starts by comparing genes between treatment groups. For each gene, a score for differential expression is calculated (for example, fold change and t-score). A gene set score is a function of scores from member genes. The gene set scores are then assigned *P*-values with either parametric or non-parametric methods.

Gene set analysis methods fall into two major categories: competitive and self-contained [9]. Competitive methods, the focus of this article, test the null hypothesis that a gene set is not more associated with the phenotype of interest than a random sample from its complement set of genes. Self-contained methods, however, test whether or not a gene set is associated with the phenotype considering only genes from the tested gene set. Under the null hypothesis, competitive methods assumes that genes are independent which is violated by gene-gene correlation observed in gene expression data. Studies have suggested the use of sample permutation to generate the null distribution for *P*-value estimation as it preserves the gene-gene correlation [10, 11]. The most popular competitive methods are based on sample permutation and competitive statistics (implicit gene sampling) for gene set scores [10, 12]. Several parametric methods have also been developed which, although faster, make strong statistical assumptions that are not always met [13–15]. While sample permutation-based approaches do not make such strong assumptions, large number of permutations are necessary for accurate *P*-value estimation. Indeed, it has recently been shown by Mishra et al. that the popular choice of 1000 permutations is inadequate and results in a loss of precision particularly visible in the tail-end of the gene set score distribution [16]. This loss of precision is important because it can result in the same *P*-value being assigned to multiple gene sets whose true significance varies. In addition to being inaccurate, the relative ranking of gene sets with the same *P*-value is arbitrary.

We recently proposed a sample permutation-based competitive gene set analysis method, mGSZ [16], that combines features from both parametric and permutation-based approaches. In mGSZ, *P*-values are estimated from empirical null distribution of gene set scores smoothed with a continuous distribution. We showed that the semi-parametric approach requires far fewer permutations (as low as 200) compared to other sample permutation-based methods and produces biologically plausible results. Despite the significant improvement in efficiency, mGSZ still requires at least six biological replicates per sample group to give accurate results. Multi-group gene expression data with fewer than six replicates, however, are common and naïve application of sample permutation in this context is, as we have stated, potentially unreliable.

This article proposes mGSZm, a new competitive gene set analysis method for multi-group gene expression data with as few as three biological replicates per sample group. The method is based on advanced sample permutation technique that utilizes all sample groups to generate an appropriate number of permutations for pairwise comparisons.

## Methods

### Permutation methods

**Fig. 1:**
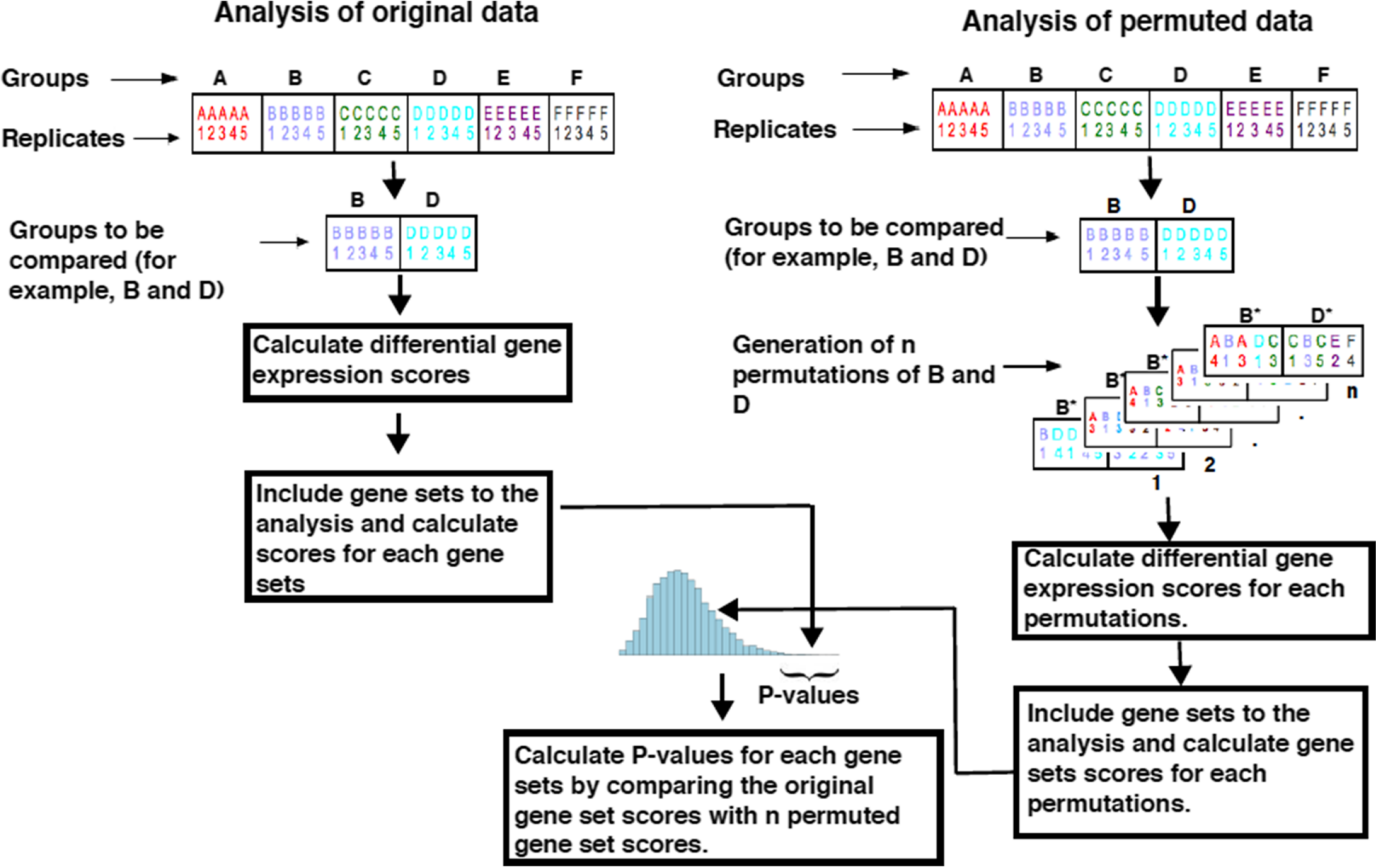
Gene set analysis pipeline. General pipeline for permutation based gene set analysis

**Fig. 2:**
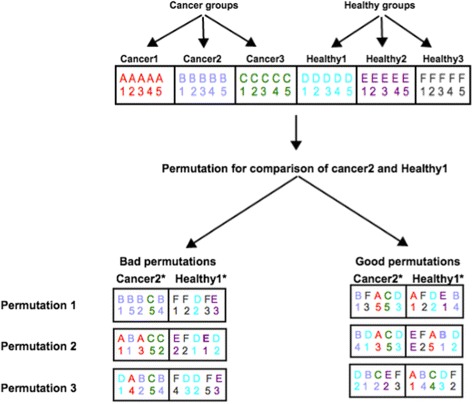
Examples of bad and good permutations. Examples of bad and good permutations. Cancer2* and Healthy1* represent permuted Cancer2 and Healthy1 groups. Under bad permutation examples, Cancer2* fails to include samples from healthy groups and Healthy1* fails to include samples from cancer groups causing strong leakage of biological signal into null distribution. Under good permutation examples, Cancer2* and Healthy1* includes samples from both healthy and cancer groups evenly

Permutation-based gene set analysis methods estimate the null distribution by recalculating gene set scores for many permutations of the data (Fig. 1). The tested gene set scores are then compared to the null distribution for the estimation of *P*-values. A large number of permutations is required to obtain the null distribution and its quality is dependent on the permutation method. An ideal null distribution would include all the important noise signals (like variations within a group and gene-gene correlations) and lack true biological signals (differences between groups). Improper permutation methods can generate permutations that are almost identical to the correct sample labels, causing true biological signal to “leak” into the null distribution (Fig. 2). This corruption of the null distribution can also occur when some groups in multi-group gene expression data are highly correlated.

We propose that, in a multi-group gene expression dataset, groups other than those being compared can be used for the estimation of null distribution. Other than the phenomena being investigated, we assume that the same inherent structure, including gene-gene correlations, occur similarly across all groups, differential gene expression score represents gene regulation and co-regulation of member genes of a gene set represents regulation of the associated biological process.

We evaluated six different permutation methods for multi-group gene expression data, referred to as Perm1-6. Perm1 is the widely used naïve permutation method of only permuting the labels of the groups being compared [10, 12, 16]. The rest of the permutation methods are new approaches proposed by us that aim to prevent signal leakage by ensuring that permuted groups do not contain many samples from a single original group. Sampling is least constrained (most random) in Perm2 and most constrained (least random) in Perm6. By constraining what permutations are generated we aim to ensure that permuted groups do not correlate with the original groups. Perm3-6 showed similar performance in our evaluations (see Additional file 1, Figure 12) and therefore, for clarity, we only describe Perm 4 in detail in this article. For details on Perm3, Perm5 and Perm6 see Additional file 1, Sections 1-4.

We use the following notation to describe each permutation method:

- Number of replicates in a group. For simplicity, we let *n* be constant for all groups.
- Total number of groups in multi-group gene expression data.
- Total number of samples in all the groups.
- Total number of samples in the groups being analyzed.
- Total number of permutations from permutation method *x*.

**Fig. 3:**
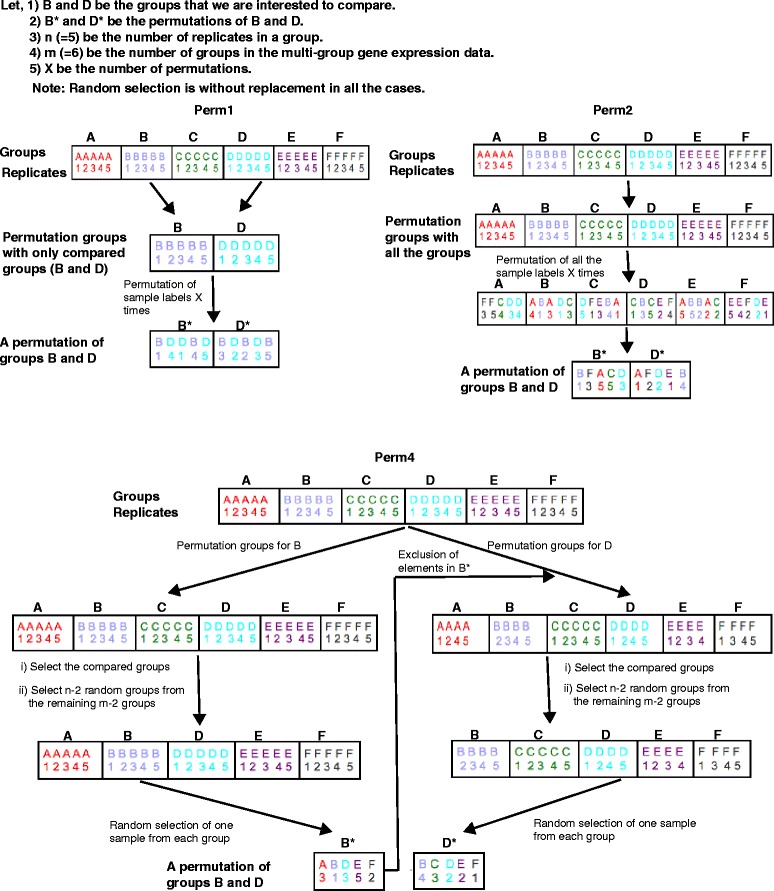
Permutation methods. Schematic diagram representing the three permutation methods (Perm1, Perm2 and Perm4). The steps shown in each of the permutation methods are repeated for X times, where X is the number of permutations. See Additional file 1, Sections 1-5 for details

**Perm1** This is the most basic permutation method where only samples from the groups being compared have their sample labels permuted (Fig. 3). This approach is not suitable for data sets with few replicates because of the limited number of unique permutations, given by:

1 $$  N|Perm1| = \frac{1}{2} \: \frac{z!}{n!(z-n)!}  $$

The factor 1/2 is to exclude permutations that are mirror images of one another and give identical results, for example, (1,1,0,0) and (0,0,1,1). For example, in multi-group gene expression dataset with *m*=6 and *n*=5, the total number of obtainable permutations is 126.

**Perm2** We permute the sample group labels for all samples in the data set, not just those being analysed. Suppose we are analysing two sample groups *B* and *D*, a single permutation is defined as the current samples with those labels (Fig. 3). This method allows for the situation where permutations may not contain a single sample from the groups currently being analysed. Furthermore, there is a risk of incorporating biological signal into the null distribution because permutations can include many or all samples from their respective original groups (Fig. 2). The total number of unique permutations from this method is given by:

2 $$  N|Perm2| = \frac{1}{2} \: \frac{y!}{n! n!(y-2n)!}  $$

The total number of unique permutations that can be obtained with our example dataset with *m*=6 and *n*=5 is 3.8*e*+9.

**Perm4** Similar to Perm2, Perm4 produces the null distribution using all sample groups in the data set. When *m*>*n*, permutations are generated in two stages: group selection followed by sample selection from those groups (Fig. 3). Groups are selected by starting with the two groups being analyzed followed by sampling *n*−2 additional groups without replacement. Samples are selected randomly, one from each of the previously selected sample groups. This procedure must be repeated to obtain the second half of the permutation, with the exception that samples found in the first half are explicitly filtered out. The mean estimate, therefore, is not strongly influenced by any individual groups. For simplicity, we will consider the lower bound of the number of unique permutations, which is given by:

3 $$ {}\begin{aligned} N|Perm4| =\! \frac{1}{2}\: n^{n} (n-1)^{n} \left(\frac{(m-2)!}{(n-2)!(m-n)!}\right)^{2}, \quad m\! >\! n \end{aligned}  $$

The total number of obtainable permutations from Perm4 for an example case with *m*=6 and *n*=5 is 2.6*e*+7

### Gene Set Z-score

We implemented all permutation methods together with Gene Set Z-score (GSZ) [17] for evaluation. GSZ is a gene set scoring method based on a hypergeometric enrichment score weighted with the differential expression test scores [16, 17] and has been successfully used in several projects [18–20]. As the scoring function is a constant, any differences in the results can be attributed to differences in the permutation methods. Based on the results of the evaluation, we implemented Perm4 in mGSZ. We refer to the modified version of mGSZ as mGSZm (Table 1).

**Table 1:** Compared competitive gene set analysis methods

Compared methods	Permutation type	Descriptions	
mGSZm	sample	mGSZ from R package *mGSZ* updated with Perm4	
mGSA	sample	Gene Set Z-score based gene set analysis method similar to GSA [17]	
Allez	parametric	Allez from R package *allez* [14]	
wKS	sample	wKS from R package *mGSZ* [16]	
GAGE	parametric	Generally applicable gene set enrichment from R package gage [21]	
CAMERA	parametric	Correlation adjusted mean rank gene set test from R package limma [13]	
QuSAGE	parametric	Quantitative set analysis of gene expression from R package qusage [22]	
romer	sample	Rotation testing using mean ranks from R package limma [24, 25]	

### Compared gene set analysis methods

We evaluated mGSZm together with several gene set analysis methods from the literature: GAGE (Generally Applicable Gene-set Enrichment) [21], CAMERA (Correlation Adjusted MEan RAnk gene set test) [13], QuSAGE (Quantitative Set Analysis of Gene Expression) [22]) and Allez [14] (Table 1). GAGE is based on parametric gene randomization and thus neglects gene-gene correlation. Another major difference between GAGE and the other methods is that GAGE is based on log fold change as gene statistics, whereas the others are based on Moderated t-test [23]. CAMERA is also based on parametric gene randomization, however it corrects the errors introduced by gene-gene correlation in a gene set by incorporating a Variance Inflation Factor (VIF) estimated directly from the data. QuSAGE is similar to CAMERA, except that it associates a complete probability density function to a gene set score instead of a *P*-value and does not require the assumption of equal variance of gene level signals in a gene set for the estimation of VIF. In Allez, the gene set scoring function calculates the mean value of the differential expression test scores for all the genes in the analyzed gene set followed by normalization. We also included the two most popular sample permutation-based competitive gene set analysis methods, mGSA and wKS. mGSA is a sample permutation-based competitive gene set analysis method that is similar to GSA (Gene Set Analysis) [10]. mGSA is based on Gene Set Z-score instead of max-mean statistics. Unlike the original GSA, mGSA is applicable for one-on-one comparison of selected groups in multi-group gene expression data. Note that we have shown improved performance of mGSA compared to GSA in our previous article [16]. wKS (weighted Kolmogorov Smirnov) is our version of gene set analysis method, GSEA [12]. In addition, we included romer [24, 25] (Rotation testing using mean ranks), a competitive gene set analysis method based on a linear model. The number of permutations in permutation based methods was set to 1000 and all the rest of the parameters in all the methods were set to default.

### Evaluation

Evaluation of gene set analysis methods is a challenge due to a lack of ground truth and the fact that any ranking of methods will vary between different datasets and evaluation criteria. While the use of simulated data is common, reliability is often questionable as it can oversimplify complex biological phenomena. Another approach is to evaluate gene set rankings based on biological relevance. This approach avoids the negative aspects of simulated data, but requires extensive literature review and assessing the relevance of gene sets is difficult. We address these challenges with three complementary evaluation approaches based on: i) data splitting, ii) detection of tissue specific gene sets and iii) generation of type 1 error.

#### Evaluation based on data splitting

**Fig. 4:**
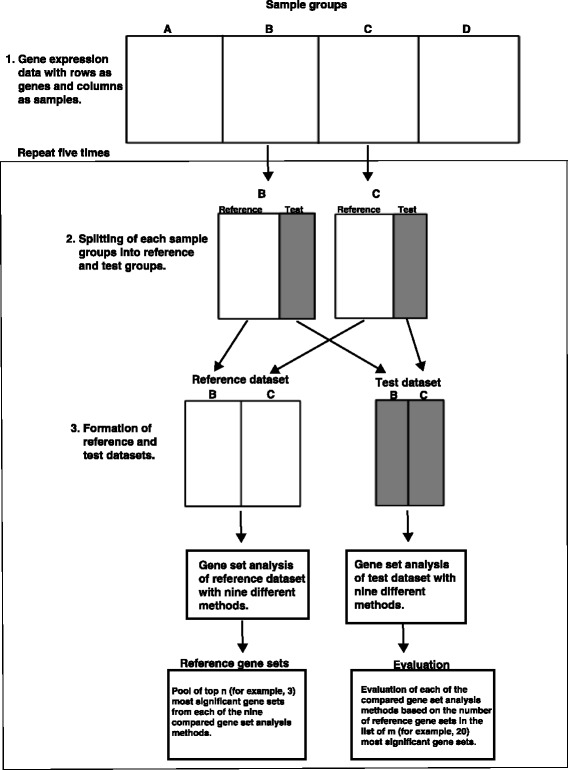
Evaluation based on data splitting. Workflow of the evaluation based on splitting of data

Our data splitting method partitions a data set into *test* and *reference* subsets (Fig. 4), similar to the cross validation approach used by Törönen et al. [17]. This approach requires a data set with more than two sample groups and a large number of replicates per group. After splitting, the test and reference partitions comprise 25% and 75% of the data (arrays), respectively. We generated *reference gene sets* by taking the union of the *n* most significant gene sets reported by each method (mGSZ, wKS, GAGE, CAMERA, QuSAGE, romer and improved versions of GSA and Allez [16]) for the reference data. We aimed to minimize any bias that the choice of *n* had on results by repeating the above procedure for multiple values of *n* (we used *n*=3,5 and 7). In the actual evaluation, we used the test subset to obtain a new ranking of significant gene sets for each method. For the top 50 gene sets, we kept a cumulative count of those also found in the reference gene set. At each rank, the average cumulative count across all values of *n* was calculated. The entire process was repeated 100 times for different data splits (20 in the evaluation of permutation methods). The graphs presented are averages over all experiments.

#### Evaluation based on detection of tissue specific gene sets

We generated a single gene set per tissue type based on tissue specific genes identified and verified by Song et al. [26]. Next for each of the six gene sets (for six tissue types), we randomly selected *x*% of genes where *x*∈(0,10,20,30,40,50,60,70,80,90) and replaced them with randomly selected genes from the remaining complementary mouse genes. This way we generated 10 tissue specific gene sets for each tissue type based on 10 different values for *x*. For example, while a gene set with 0% random genes would contain only tissue specific genes, a gene set with 90% random genes would contain only 10% tissue specific genes. So, in total we had 60 gene sets that we consider “relevant gene sets”. The relevant gene sets were then mixed with randomized gene sets. The idea was then to see which methods rank the relevant gene sets higher than the random gene sets. Note that for each pairwise comparison of two tissue types, there are 20 relevant gene sets (10 tissue specific gene sets for each tissue type). All the possible 15 pairwise comparisons of six tissue samples were done. Methods were evaluated based on average number of relevant gene sets reported in the top rank of the gene set list in all the 15 pairwise comparisons. We also evaluated the methods based on precision-recall area under curve.

#### Type 1 error test

Perm4 is a modified and more controlled version of the naïve permutation method. It is important to test whether the modification generates false positive results (type 1 error). Null gene expression data was generated by randomizing the sample labels of breast cancer gene expression data. mGSZm and all other methods except Allez were applied to the null gene expression data and the distribution of estimated *P*-values was compared. Allez was not included in this evaluation because it does not report *P*-values. *P*-values for QuSASE were calculated using pdf.pVal function in QuSAGE’s Bioconductor package. In principle, *P*-values estimated by mGSZm or similar methods based on null gene expression data with no true differential gene expression should follow a uniform distribution.

#### Asymptotic validation of Perm4

In addition to the evaluation tests described above, we tested the validity of Perm4 by comparing the null distributions of gene set scores with 10000 permutations generated with Perm1 and Perm4 using the breast cancer dataset. The groups with 33 and 23 biological replicates were compared. Given sufficient number of replicates, Perm1 is the most optimal permutation method. The idea is to test if Perm4 generates results similar to that of Perm1 with an ideal dataset. We fitted an Extreme Value distribution to the null distributions of each of the gene sets generated by Perm1 and Perm4. The Extreme Value distribution was shown to be suitable for modeling GSZ scores under the null hypothesis (Mishra et al., 2014). The similarity between the two permutation methods was measured with correlation and mean relative error (abs($\hat {x_{i}}$- *x*_*i*_)/abs(*x*_*i*_), where $\hat {x_{i}}$ and *x*_*i*_ are the parameters of the *i*th gene set from Perm4 and Perm1 respectively) between the estimated parameters (scale and location) across all the analyzed gene sets.

### Data sets

**Gene expression data** Evaluation of the methods was based on three different multi-group gene expression data sets; 1) Human primary cell data, 2) Breast cancer data, and 3) Mouse tissue gene expression data (see Additional file 1, Section 7 for details). Human primary cell and breast cancer data were used for evaluation based on data splitting. Only those sample groups that have at least 15 biological replicates, are well clustered and have no outliers were considered. Mouse tissue data was used for evaluation based on detection of tissue specific gene sets.

**Gene set data** C2 curated gene sets from the Molecular Signatures Database were used throughout [12, 27].

## Results

### Overview of the results

Table 2 summarizes the results obtained from evaluation of the permutation methods and mGSZm. Advanced permutation methods showed clearly better results compared to the naïve method, Perm1, with both datasets. mGSZm, gene set analysis method based on advanced permutation method, Perm4 showed the best overall result as compared to other gene set analysis methods across the three different evaluations with three different real biological datasets.

**Table 2:** Overview of the results

Evaluation approaches	Evaluated methods	Principles	Data	Results
Data splitting (cross-validation type)	Permutation methods	Gene expression data was splitted into two parts; reference and test. The methods were evaluated based on their ability to replicate results from reference dataset using test dataset.	1. Human primary cell data. 2. Breast cancer data.	Perm1 showed bad performance as compared to the rest of the permutation methods in both datasets.
Data splitting (cross-validation type)	mGSZm and seven other gene set analysis methods shown in Table 1.	Same as above	1. Human primary cell data. 2. Breast cancer data.	mGSZm ranked the maximum number of*reference gene sets* in the list of top 50 gene sets from test data in both datasets.
Detection of tissue specific gene sets in tissue gene expression data	mGSZm and seven other methods shown in Table 1.	Method that ranked maximum number of tissue specific gene sets on the top 50 gene sets list was considered the best	Mouse tissue gene expression data.	mGSZm ranked the maximum number of tissue specific gene sets in the list of top 50 gene sets.
Type 1 error test	mGSZm and seven other methods shown in Table 1.	An ideal method is the one that generates uniform distribution of gene set score *p*-values obtained from null gene expression data.	Breast cancer data.	mGSZm showed slightly left skewed null *p*-value distribution. Similar results were obtained with other methods.

### Evaluation of permutation methods

**Fig. 5:**
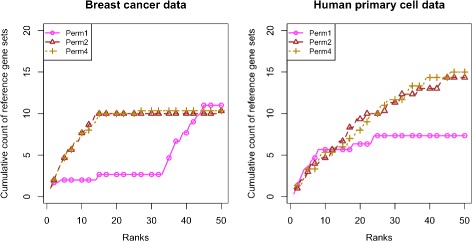
Permutation methods evaluation plot. *Reference gene sets* identified by the three gene set analysis methods based on three different permutation methods with two different datasets; 1) Breast cancer data, 2) Human primary cell data. Figures represent cumulative count of *reference gene sets* over the ranked list of top 50 gene sets from *test* data reported by each of the methods

Evaluation of the permutation methods was based on data splitting approach with two different datasets. Perm1, the naïve permutation method was the worst performing method with both datasets (Fig. 5). Perm4 reported ∼ 8 (average over 20 different data splits) more relevant gene sets at rank positions 27 to 33 in breast cancer data and 46 to 50 in primary cell data. Perm2 showed similar results, however, we prefer Perm4 to Perm2 because Perm2 is the least constrained permutation method and it cannot prevent biological signal leakage into the null distribution.

### Evaluation of mGSZm

#### Detection of reference gene sets

**Fig. 6:**
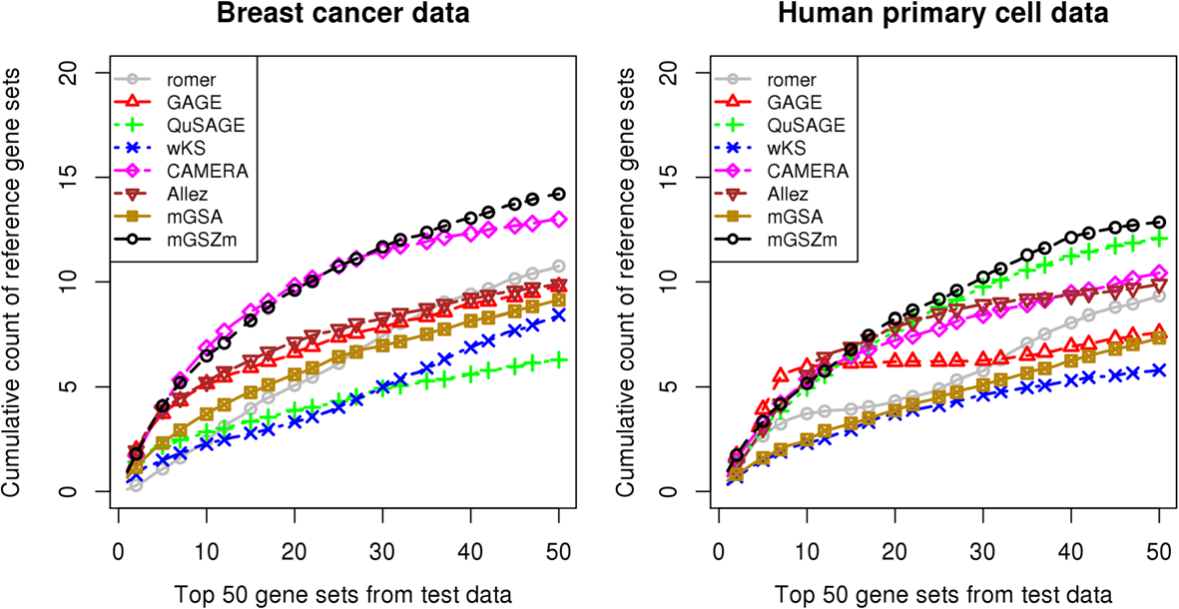
mGSZm evaluation based on data splitting *Reference gene sets* identified by the eight compared gene set analysis methods with two different datasets; 1) Breast cancer data, 2) Human primary cell data. Figures represent cumulative count of *reference gene sets* over the ranked list of top 50 gene sets from *test* data reported by each of the compared methods

**Fig. 7:**
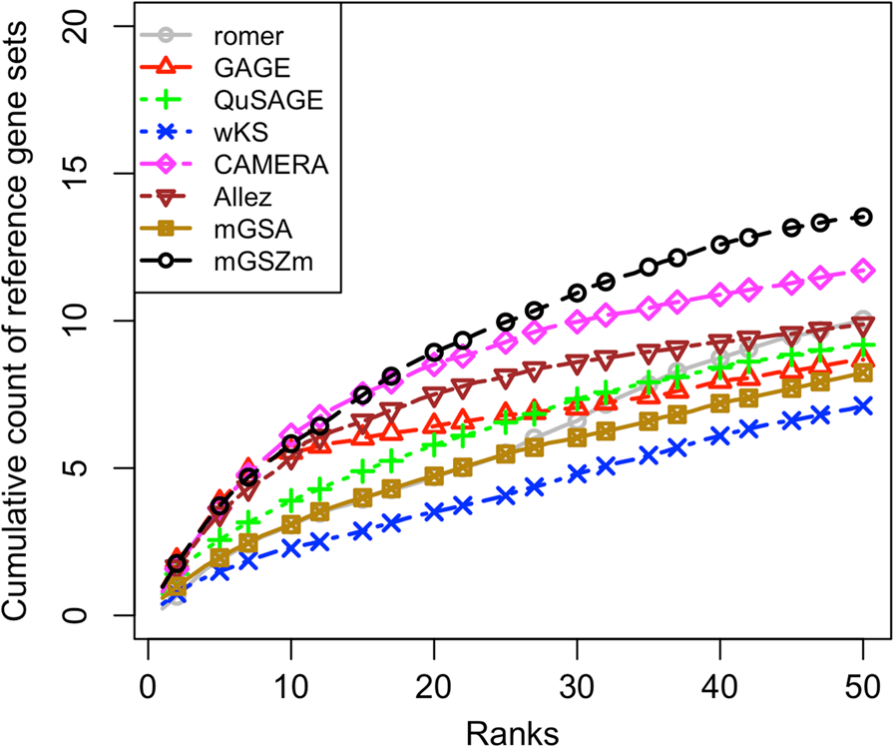
mGSZm evaluation based on average of results from two datasets. Average number of *reference gene sets* identified by the eight compared gene set analysis methods with two different datasets; 1) Breast cancer data, 2) Human primary cell data. Figures represent average of cumulative counts of *reference gene sets* over the ranked list of top 50 gene sets from *test* data reported by each of the compared methods

mGSZm reported the maximum number of reference gene sets in both datasets (Fig. 6). Note the inconsistencies in the performance of CAMERA, QuSAGE, GAGE and Allez in the evaluations using the two datasets. While CAMERA is the closest competitor to mGSZm in breast cancer data with mGSZm leading only by about one gene set (average over 100 different data splits) at rank positions 37 to 50, it reports about two gene sets less at rank positions 33 to 50 in primary cell data. Similarly, GAGE and Allez reported about one gene set more than mGSZm at rank positions 4 to 15 in primary cell data. However, the performance of the methods dropped clearly at rest of the rank positions in the same dataset and all the rank positions in breast cancer dataset. While QuSAGE is the closest competitor to mGSZm in primary cell data, it is one of the worst performing method in case of breast cancer data. Overall best performance of mGSZm is clearer in the plot of average cumulative counts of the two datasets where mGSZm leads the second best method CAMERA by about 2 gene sets over the rank positions 38 to 50 (Fig. 7).

#### Detection of tissue specific gene sets

**Fig. 8:**
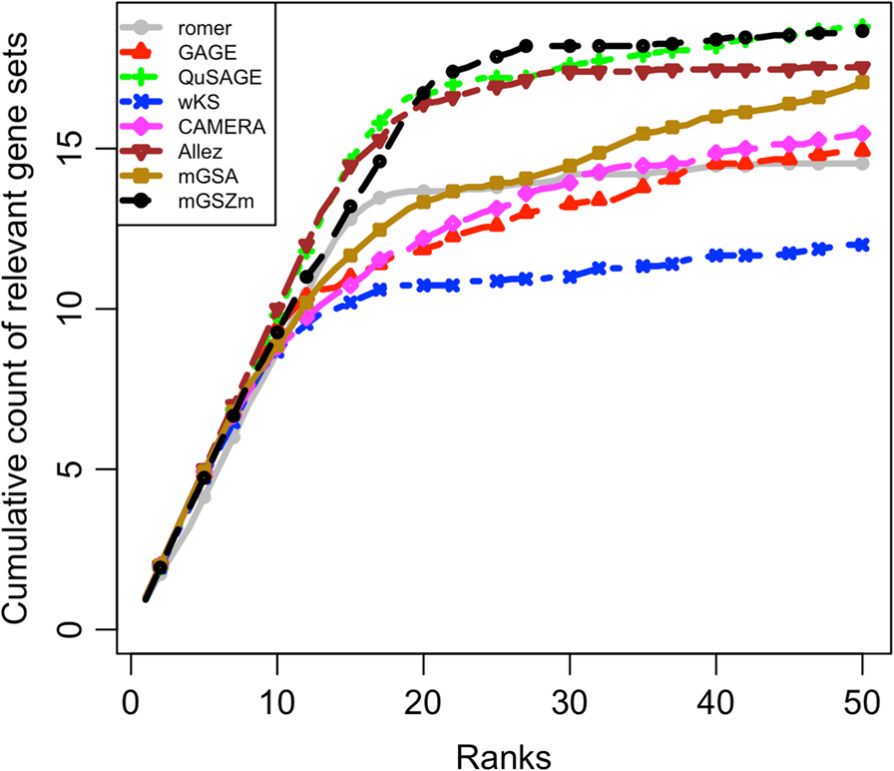
Evaluation based on tissue specific gene sets Tissue specific gene sets identified by the eight compared gene set analysis methods. Figure represents average cumulative count of tissue specific gene sets over the ranked list of top 50 gene sets reported by each of the compared methods in 15 pairwise comparisons of six different tissue samples

mGSZm showed the best overall performance (Fig. 8). Note that CAMERA, which was a close competitor to mGSZm in other evaluations is one of the worst performer in this evaluation identifying about five gene sets less than mGSZm at rank positions 20 to 27. Further, Allez and QuSAGE, which are close competitors to mGSZm in this evaluation performed weakly in other evaluations (Fig. 7). For the results from individual pairwise comparisons, see Additional file 1, Section 8. We also evaluated the compared methods based on precision-recall area under curve (precision-recall AUC) for 13 of the 15 pairwise comparisons. The exclusion of two of the pairwise comparisons was due to ties in *p*-values obtained from mGSZm because of high biological signal. Also, romer was not included in the precision-recall AUC based evaluation because the romer function in R package reports *p*-values with multiple ties. Ties in *p*-values are common also in *p*-values obtained from wKS. However, we use wKS gene set scores (instead of *p*-values) and include wKS in the evaluation. Based on the evaluation, mGSZm, mGSA, Allez, wKS and QuSAGE showed similarly superior performance over CAMERA and GAGE in 12 of the 13 pairwise comparisons (see Additional file 1, Section 8).

#### Generation of type 1 error

**Fig. 9:**
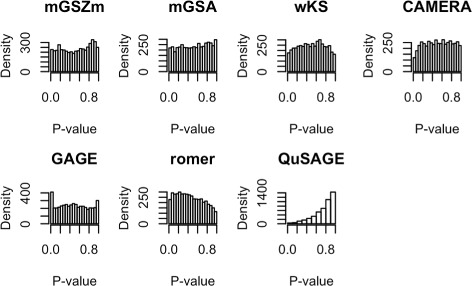
Histogram of *p*-values. Histogram of *p*-values obtained from mGSZm and six other compared gene set analysis methods with null gene expression data with no true differential gene expression

mGSZm produces a slightly left skewed, but approximately uniform distribution of *P*-values, showing mGSZm to be fairly conservative in generation of type 1 error (Fig. 9). *P*-value distributions for most other methods showed similiar distributions, with the exception of QuSAGE and romer. QuSAGE is heavily left skewed and romer is slightly right skewed.

#### Asymptotic validity of Perm4

Our results show that, across all the analyzed gene sets the correlations of the estimated parameters, scale and location were 0.99 and 0.98, respectively, and the mean relative errors between the parameters were 0.04 and 0.07, respectively.

## Discussion

We presented a novel competitive gene set analysis method based on advanced sample permutation for multi-group gene expression data with as few as three replicates. Our results show that the naïve permutation method commonly used in other methods should not be used with the type of data set in question. In addition, our results emphasize the importance of using a multi-faceted evaluation approach, without which, we would be unable to assess the relative ranking of each method.

The permutation methods considered in this work are different from each other in the way they model the background signal of the data. Our results show that naïve sample permutation (Perm1) is unreliable for the size of data set in question and, further, that generating permutations using samples drawn from the complete data set can significantly improve results (Figs. 7, 8). Exactly how these permutations should be generated, however, is a deeper question than expected. Perm2, for example, uses all sample groups, but can group highly correlated samples and promote false negatives. Perm2 is therefore unsuitable for gene expression data with highly correlated sample groups. The proposed permutation method, Perm4, outperforms the other methods because it can generate sufficient number of permutations while controlling leakage of biological signals into the null distribution. Perm4 can be used in any sample permutation based gene set analysis of multi-group gene expression data irrespective of platform, i.e. microarray or RNAseq. However, we strongly recommend at least four replicates whenever possible.

mGSZm is based on Perm4 and Gene Set Z-score [17]. Further, mGSZm is based on an asymptotic method for *P*-value estimation instead of an empirical method [16]. The use of asymptotic, rather than empirical, *P*-values requires fewer permutations and thus speeds up the analysis process without compromising accuracy. We have shown in our previous article [17] that asymptotic *P*-values calculated with 500 permutations are as accurate as empirical *P*-values calculated with 100,000 permutations. Thus, mGSZm has an advantage that allows for more accurate ranking of gene sets compared to other methods, like mGSA and wKS, that use empirical *P*-values. The better performance of mGSZm compared to the parametric methods could be because they are based on strong statistical assumptions which are not always met. Indeed, all the parametric methods showed inconsistent performance across data sets and evaluation criteria.

To understand why some methods performed inconsistently between data sets, we investigated whether there was a bias towards gene sets of a particular size or differential gene expression signal level. We noticed that in the breast cancer data, a large proportion (about 40%) of reference gene sets include over 100 genes, whereas in human primary cell data, there are fewer reference gene sets (about 20%) with over 100 genes. The mouse tissue specific gene sets contained, on average, 10 genes. Another difference between the data sets is that separation between sample groups is weaker in breast cancer data as compared to the other datasets. It is evident that, in contrast to other methods, mGSZm showed consistent performance irrespective of these variables. With regard to the performance of other methods, we speculate that 1) CAMERA appears to favor larger gene sets as it failed slightly in primary data and significantly in mouse data, 2) QuSAGE seems to prefer stronger expression signal as it performed well with primary human cell and mouse data, and 3) Allez performed well on the mouse data and therefore probably favors smaller gene sets. This highlights the importance of using multiple data sets when comparing different methods.

## Conclusion

We present mGSZm, a method for gene set analysis of multi-group gene expression data with as few as three replicates. Our proposed permutation method maintains the correlation structure of the genes and permutes samples such that leakage of biological signal into the null distribution is prevented.

## Additional file

Additional file 1 This file contains supplementary figures and detailed descriptions of methods. (PDF 4540 Kb)

## Abbreviations

AUC: Area under curve
CAMERA: Correlation adjusted mean rank gene set test
GAGE: Generally applicable gene-set enrichment
GSA: Gene set analysis
GSEA: Gene set enrichment analysis
GSZ: Gene set z-score
mGSA: Modified gene set analysis
mGSZ: Modified gene set z-score
mGSZm: Modified gene set z-score for multi-group gene expression data
QuSAGE: Quantitative set analysis of gene expression
romer: Rotation testing using mean ranks
VIF: Variance inflation factor
wKS: Weighted Kolmogorov-Smirnov
