# Supplementary material for: Robust multi-group gene set analysis with few replicates
Source: BMC Bioinformatics. 2016 Dec 9;17:526. doi: 10.1186/s12859-016-1403-0 (PMC5148902; doi:10.1186/s12859-016-1403-0)
Supplement: Additional file 1 — This file contains supplementary figures and detailed descriptions of methods. (PDF 4540 Kb) [file 12859_2016_1403_MOESM1_ESM.pdf]

Supplementary materials
Robust multi-group gene set analysis with few replicates

Pashupati Mishra, Alan Medlar, Liisa Holm, Petri Törönen

Contents

1 Perm3 **2**

2 Perm4 **5**

3 Perm5 **9**

4 Perm6 **10**

5 Evaluation of permutation methods **11**

6 Permutation in exceptional cases **12**

7 Datasets **14**

8 Evaluation based on detection of tissue specific gene sets **14**

8.1 Cumulative count plots 14

8.2 Precision-recall curves 14

1 Perm3

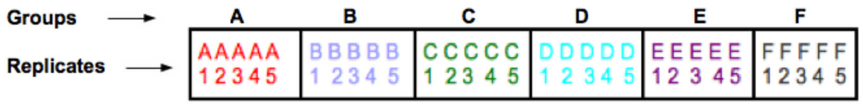

Figure 1: Sample labels of multi-group gene expression data with six groups and five replicates per group.

Lets consider a gene expression dataset with m sample groups and n replicates per sample group. In order to simplify the illustration of the permutation methods, lets consider the sample labels of the gene expression data as shown in Figure 1.

For simplicity, lets consider that n is constant for all the sample groups.

When $m > n$*,*

Lets consider that we are interested to compare groups B and D in Figure 1.

Permutation of group B,

The permutation groups for group B constitutes all the sample groups of the dataset and thus is same as the original sample labels (Figure 1).

The first step is group selection which involves random selection (without replacement) of n groups from the permutation groups containing m groups (Figure 2). Note that all the random selections in this article are without replacement.

$$\text{Number of ways to select } n \text{ groups from } m \text{ groups} = \left(\frac{m!}{n!(m-n)!}\right) \tag{1}$$

Next step is random selection of one sample from each of the selected groups (Figure 3).

$$\text{Number of ways to select } n \text{ samples from } n \text{ groups} = n^n \tag{2}$$

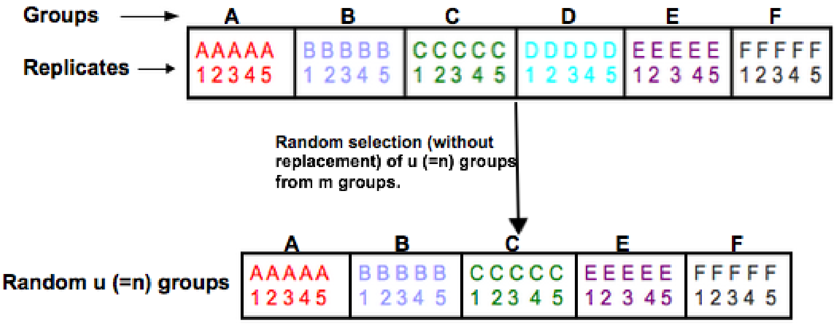

Figure 2: Random selection of n groups from m groups.

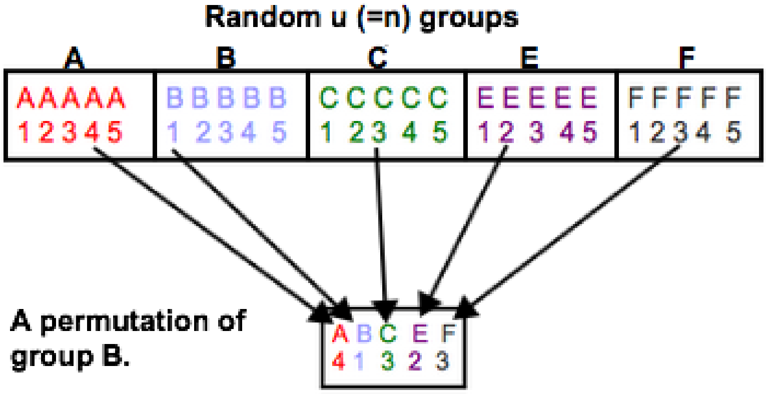

Figure 3: Random selection of one sample from each of the selected groups.

Permutation of group D

The permutation group for group D constitutes all the sample groups of the dataset after filtering out the elements in permuted group B (Figure 4). Thus, n out of m groups in the permutation groups for group D contain $(n-1)$ elements. However, for simplicity, we consider $(n-1)$ elements in all the m groups.

The first step is random selection of n groups from the permutation groups which consists of m groups (Figure 5).

$$\text{Number of ways to select } n \text{ from } m = \left(\frac{m!}{n!(m-n)!}\right) \tag{3}$$

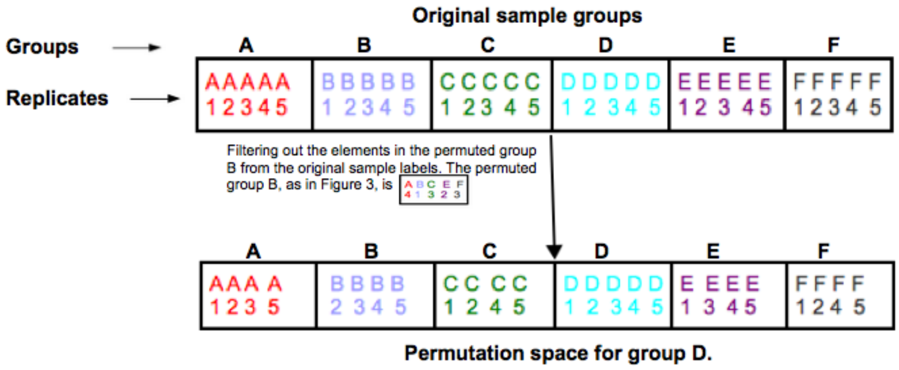

Figure 4: Filtering out of elements in permuted group B from the original sample labels to generate permutation groups for group D.

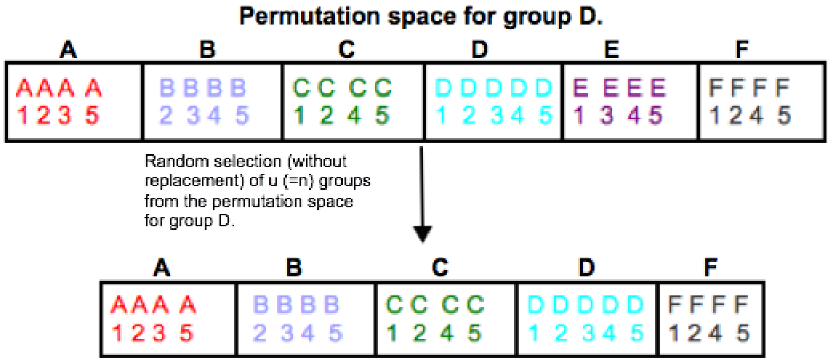

Figure 5: Random selection of n groups from the permutation groups for group D.

Next step is random selection of one sample from each of the selected groups (Figure 6).

$$\text{Number of ways to select } n \text{ samples from } n \text{ groups} = (n-1)^n \tag{4}$$

The total number of obtainable permutations from Perm3 is given by multiplying expressions on right hand side of the Equations 1, 2, 3, and 4,

$$N|Perm3| = \left(\frac{m!}{n!(m-n)!}\right)^2 .n^n.(n-1)^n.\frac{1}{2} \tag{5}$$

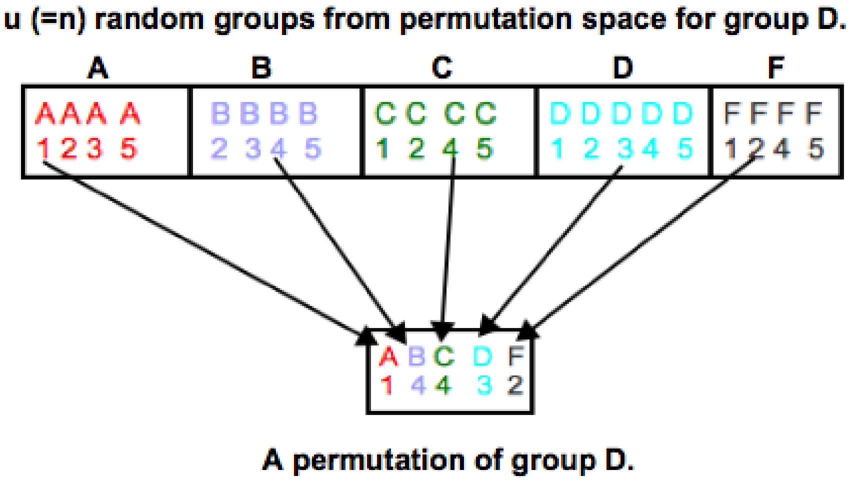

Figure 6: Random selection of one sample from each of the selected groups.

The factor 1/2 in Equation 5 is to avoid permutations that are mirror image of one another (for example, (1,1,0,0) and (0,0,1,1)).

2 Perm4

We consider the same notation and dataset example as in Section 1.

When $m > n$,

Here again, lets consider that we are interested to compare groups B and D.

Permutation of group B,

The permutation groups for group B constitutes all the sample groups of the dataset (Figure 1).

The first step is to select the pair of groups being compared (B and D) and then to randomly select $(n-2)$ groups from the remaining $(m-2)$ groups (Figure 7). Lets call the resulting set of groups as "groups for permutation of B".

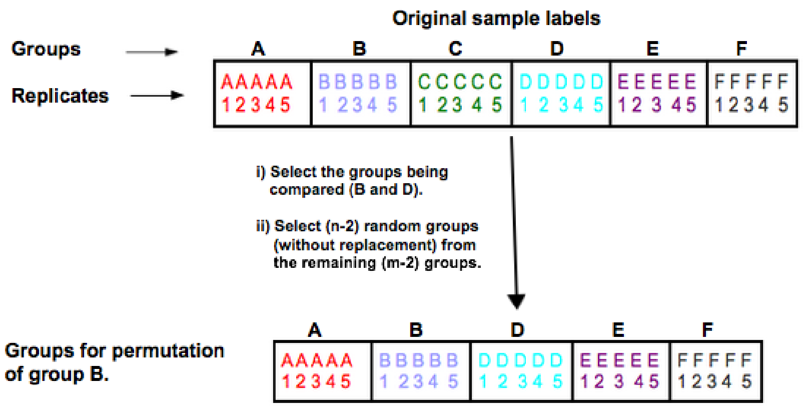

Figure 7: Generation of groups for permutation of B.

$$\text{Number of ways to select } (n-2) \text{ groups from } (m-2) \text{ groups} = \left(\frac{(m-2)!}{(n-2)!(m-n)!}\right) \quad (6)$$

Thus, the total number of groups will be, group B + group D + 3 random groups = n

Next, step involves random selection of one sample from each of the selected groups (Figure 8).

$$\text{Number of ways to select } n \text{ samples from } n \text{ groups} = n^n \quad (7)$$

Permutation of group D

The permutation groups for D constitutes all the sample groups of the dataset after filtering out the elements in permuted group B (Figure 9). Thus, n out of m groups in the permutation space for group D contain $(n-1)$ elements. However, for simplicity, we consider $(n-1)$ elements in all the m groups.

The first step is to select the pair of groups being compared (groups B and D) and then to randomly select $(n-2)$ groups from the remaining $(m-2)$ groups (Figure 10).

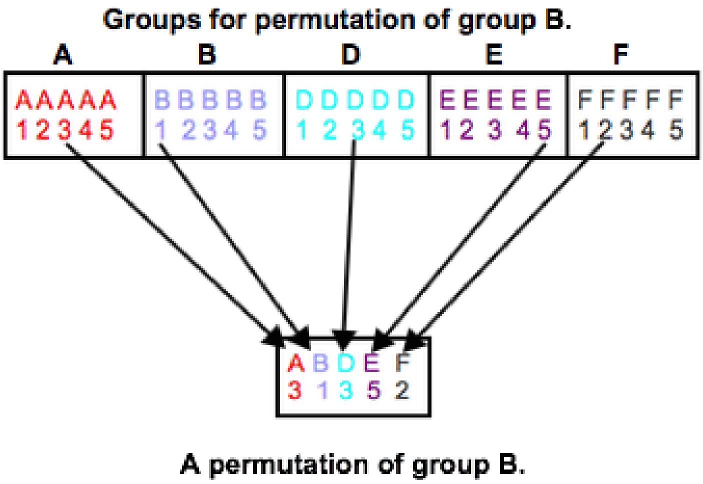

Figure 8: Random selection of one sample from each of the selected groups.

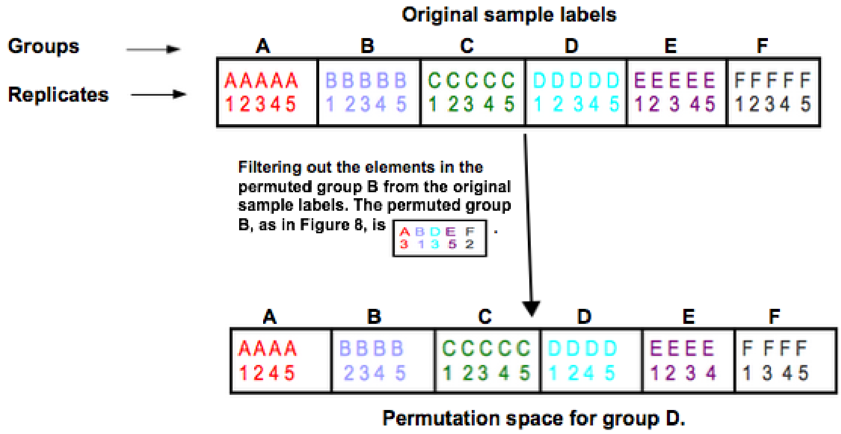

Figure 9: Filtering out of elements in permuted group B from the original sample labels to generate permutation groups for D.

$$\text{Number of ways to select } (n-2) \text{ groups from } (m-2) \text{ groups} = \left(\frac{(m-2)!}{(n-2)!(m-n)!}\right) \quad (8)$$

Thus, the total number of groups will be group B + group D + 3 random groups = n

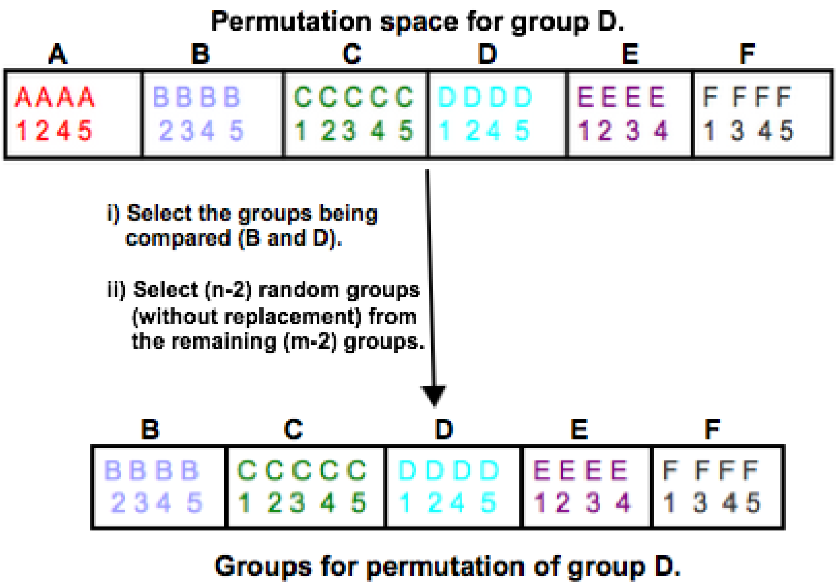

Figure 10: Generation of groups for permutation of D.

Next step is to randomly select one sample from each of the selected groups (Figure 11).

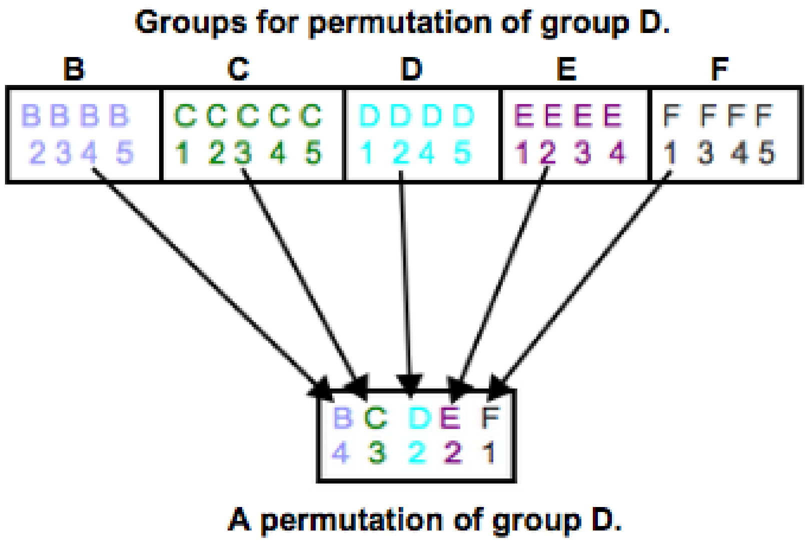

Figure 11: Random selection of one sample from each of the selected groups.

$$\text{Number of ways to select } n \text{ samples from } n \text{ groups} = (n-1)^n \tag{9}$$

The total number of obtainable permutations from Perm4 is given by multiplying expressions on right hand side of the Equations 6, 7, 8, and 9,

$$N|Perm4| = \left(\frac{(m-2)!}{(n-2)!(m-n)!}\right)^2 .n^n.(n-1)^n.\frac{1}{2} \tag{10}$$

The factor 1/2 in Equation 10 is to avoid permutations that are mirror image of one another (for example, (1,1,0,0) and (0,0,1,1)).

3 Perm5

We consider the same notation and dataset example as in Section 1.
When $m > n$,

Lets consider that we are interested to compare groups B and D.

Perm5 is similar to Perm3 except that the permutation groups for group D is restricted to only those groups which were used for permuting group B.

Permutation

The permutation group for group B constitutes all the sample groups of the dataset.

The first step is to randomly select n groups from the permutation groups which consists of m groups.

$$\text{Number of ways to select } n \text{ from } m = \left(\frac{m!}{n!(m-n)!}\right) \tag{11}$$

Next step is to randomly select pair of samples from each of the selected groups and distribute each of the pair of samples to groups B and D.

$$\text{Number of ways to select n pair of samples from } n \text{ groups} = n^n.(n-1)^n \tag{12}$$

The total number of obtainable permutations from Perm5 is given by multiplying expressions on right hand side of the Equations 11 and 12.

$$N|Perm5| = \frac{m!}{n!(m-n)!}.n^n.(n-1)^n.\frac{1}{2} \quad , m > n \tag{13}$$

4 Perm6

We consider the same notation and example dataset as in Section 1.
When $m > n$,

Lets consider that we are interested to compare groups B and D.

Perm6 is similar to Perm4 except that the permutation groups for D is restricted to only those groups which were used for permuting group B (Figure 1 in the main article).
Permutation

The permutation space for group B constitutes all the sample groups of the dataset.

The first step is to select the pair of groups being compared (groups B and D) and then to randomly select $(n-2)$ groups from the permutation space which consists of the remaining $(m-2)$ groups.

$$\text{Number of ways to select } (n-2) \text{ groups from } (m-2) \text{ groups} = \left(\frac{(m-2)!}{(n-2)!(m-n)!}\right) \tag{14}$$

Thus, the total number of groups will be, group B + group D + 3 random groups = k

Next step is to randomly select pair of samples from each of the selected groups and distribute each of the pair of samples to groups B and D.

$$\text{Number of ways to select n pair of samples from } n \text{ groups} = n^n.(n-1)^n \tag{15}$$

The total number of obtainable permutations from Perm6 is given by multiplying expressions on right hand side of the Equations 14 and 15.

$$N|Perm6| = \frac{(m-2)!}{(n-2)!(m-n)!}.n^n.(n-1)^n.\frac{1}{2} \quad , m > n \tag{16}$$

5 Evaluation of permutation methods

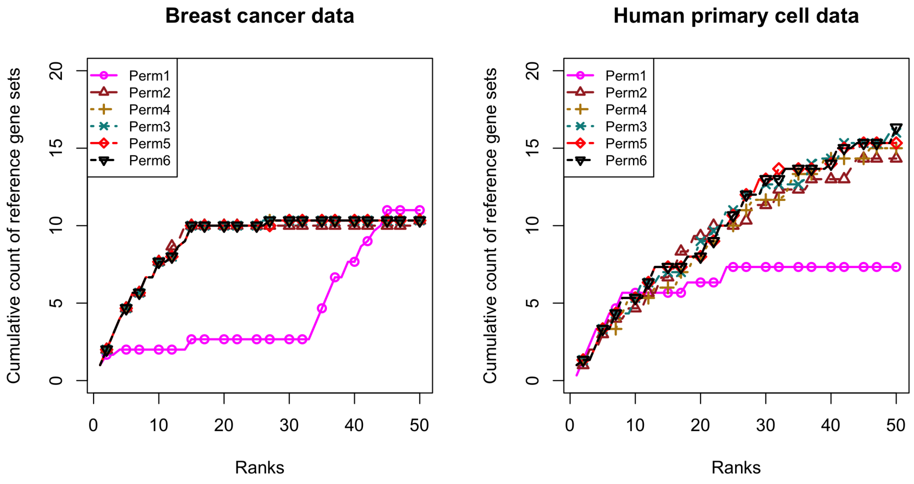

Figure 12: *Reference gene sets* identified by the six gene set analysis methods based on six different permutation methods with two different datasets; 1) Breast cancer data, 2) Human primary cell data. Figures represent cumulative count of *reference gene sets* over the ranked list of top 50 gene sets from *test* data reported by each of the compared methods.

6 Permutation in exceptional cases

The derivations in Sections 1, 2, 3, and 4 are based on the cases where $m > n$, where m is the total number of sample groups in the dataset and n is the number of replicates in a sample group. For simplicity, we considered that n is constant for all the sample groups in the dataset. Perm3-6 are same when $m = n$ or $m < n$.

When $m = n$,

Using the same notation as in the previous sections, the permutation groups for both group B and group D consists of all the sample groups in the dataset.

Permutation of group B and group D is done by randomly selecting pair of samples from each of the permutation groups and distribute each of the pair of samples to group B and group D.

Thus, the total number of obtainable permutations is given by,

$$N|p| = n^n.(n-1)^n.\frac{1}{2} \tag{17}$$

When $m < n$,

If $m < n$, pair of samples, one for each of the analyzed groups (group B and group D), is sampled from each of the permutation groups. The permutation groups are updated by filtering out the items that has been used already and the sampling process is repeated until n items for each of the analyzed groups have been sampled. Total number of obtainable permutations in this case is given by,

$$N|Perm3''| = \prod_{i=1}^{n} \frac{m!}{n_i!n_i!(m-2n_i)!}.\frac{1}{2} \tag{18}$$

where, n_i is the number of items to be sampled from i_{th} group.

When n is variable among sample groups,

In a situation where n is variable among sample groups, let x and y be the number of samples in the compared groups B and D and $x > y$. Then, pair of items, one for each of

the compared groups, is sampled from each of the groups in the permutation groups. The permutation groups are updated by filtering out the items that has been used already and the sampling process is repeated with groups with at least two samples until x items for each of the compared groups have been sampled. In case if none of the groups in the updated permutation space has at least two samples, the groups in the permutation space are merged and remaining of the x items in the compared groups are sampled from the merged groups. Permutations of the compared group with y items is then generated by randomly drawing y samples from the set of x items.

7 Datasets

Human primary cell data, Human primary cell gene expression data was downloaded from GSE49910. A total of 124 arrays of Affymetrix Human Genome U133 Plus 2.0 expression arrays was downloaded. The dataset consists of eight sample groups of different cell types - embryonic stem cells, tissue stem cells, epithelial cells, fibroblasts, endothelial cells, osteoblasts, keratinocytes and smooth muscle cells. The evaluation of the methods was based on analysis of the sample groups that have at least 15 biological replicates, are well clustered and have no outliers (endothelial cells and keratinocytes).

Breast cancer data, The data was downloaded from GEO series GSE3165. The data includes 94 arrays of platform GPL887 (Agilent Human 1A Microarray V2) with six sample groups corresponding to six molecular subtypes of breast cancer. The subtypes are basal-like, luminal A, luminal B, Her2, normal-like and claudin-low. The evaluation of the methods was based on comparison of the sample groups that have at least 15 biological replicates, are well clustered and have no outliers (Basal-like and Her2).

Mouse tissue gene expression data, Affymetrix Mouse Genome 430 2.0 Array expression profiles from six mouse tissues (kidney, liver, lung, heart, muscle and adipose) were downloaded from GEO DataSet (GDS3142). The dataset consists of four replicates for kidney and three replicates for each of the rest of the tissues.

8 Evaluation based on detection of tissue specific gene sets

8.1 Cumulative count plots

In this section, we show the plots representing individual pairwise comparisons of the six tissue samples in mouse tissue specific data (see Results from the main article). The plots represent cumulative counts of tissue specific gene sets over the ranked list of top 50 gene sets reported by each of the compared methods with mouse tissue specific gene expression data.

8.2 Precision-recall curves

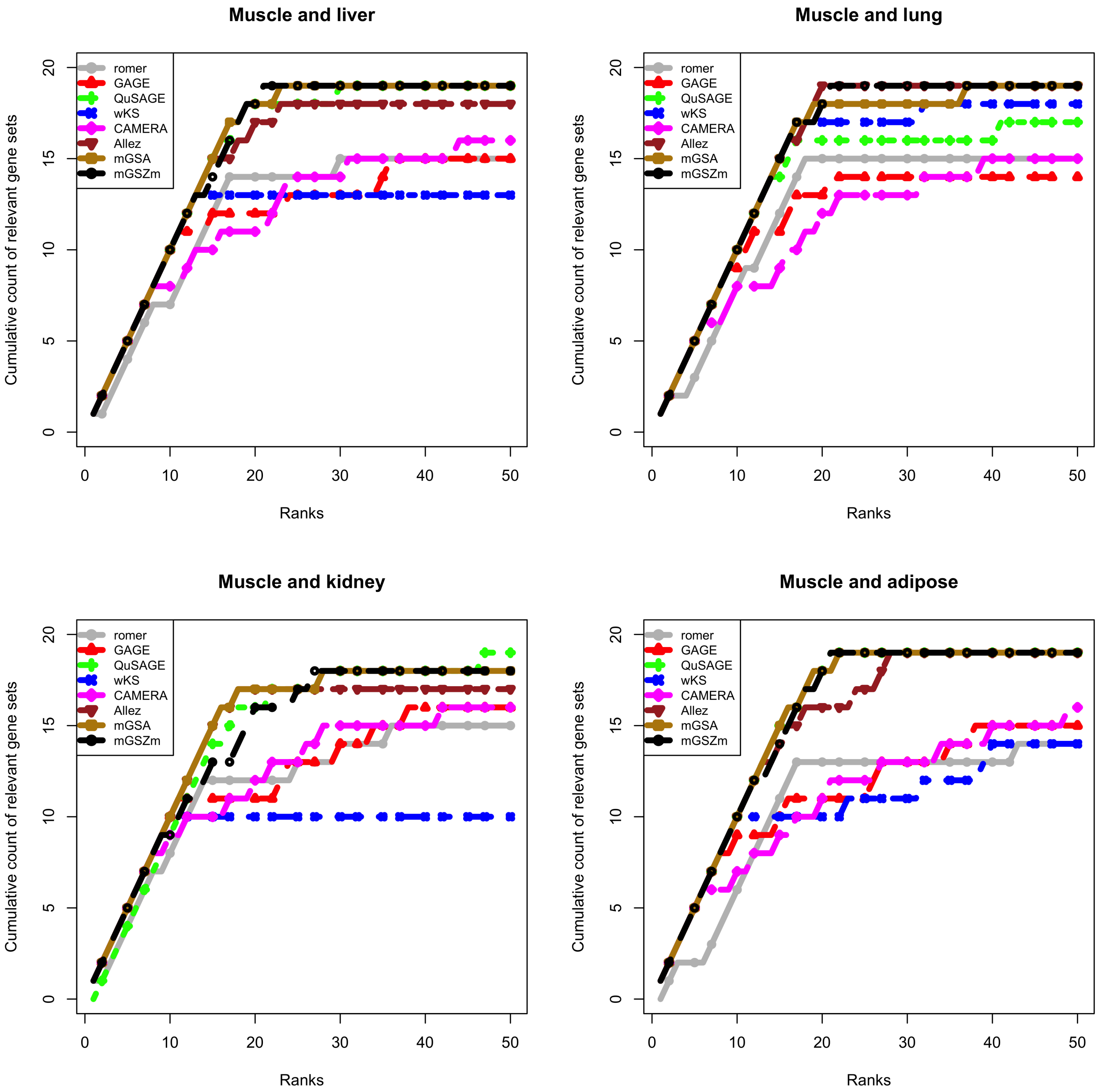
Muscle and liver
Muscle and lung
Muscle and kidney
Muscle and adipose
Cumulative count of relevant gene sets
Ranks
0
5
10
15
20
30
40
50
romer
GAGE
QuSAGE
wKS
CAMERA
Allez
mGSA
mGSZm

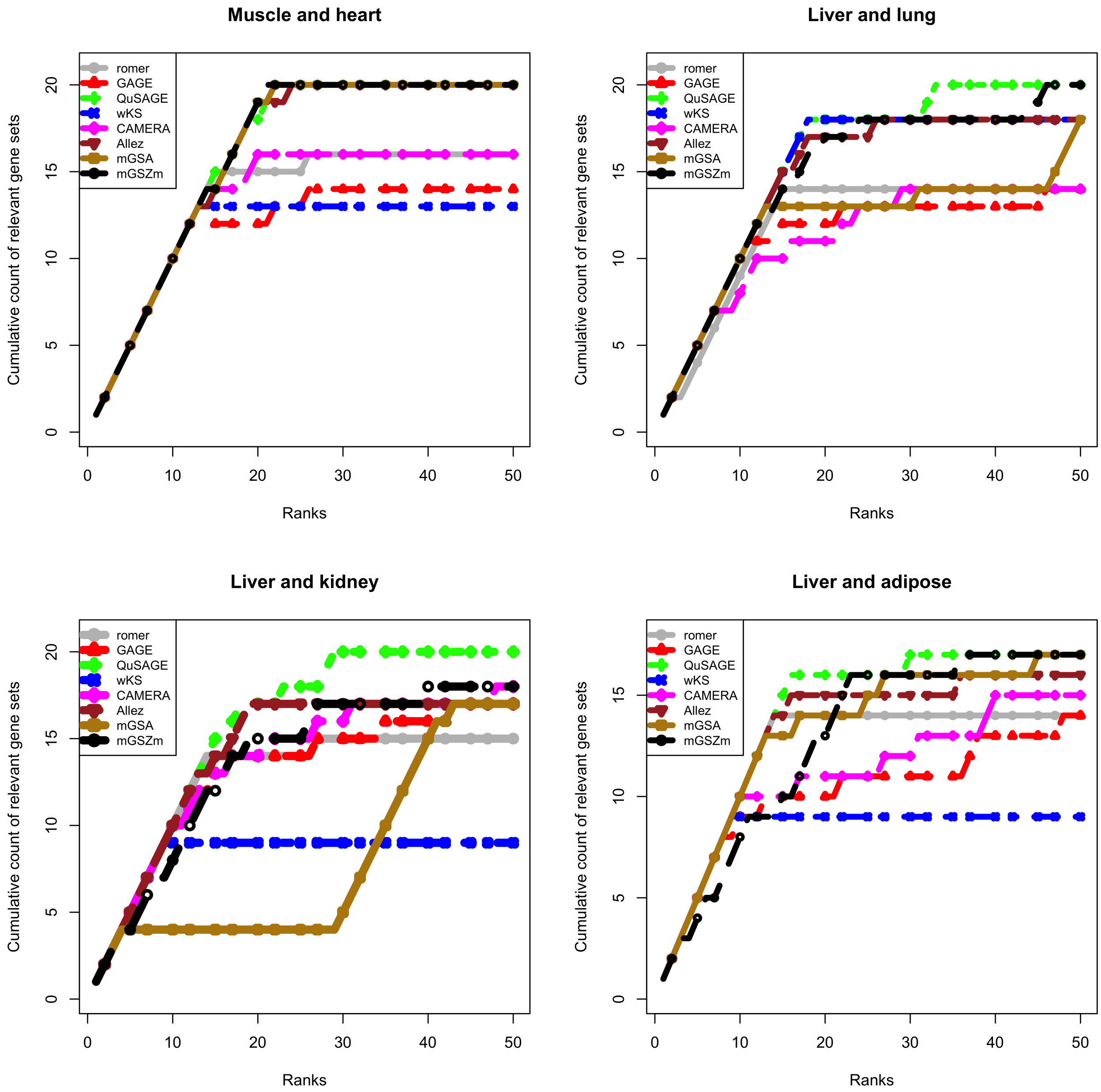
Muscle and heart
Liver and lung
Liver and kidney
Liver and adipose
romer
GAGE
QuSAGE
wKS
CAMERA
Allez
mGSA
mGSZm
Cumulative count of relevant gene sets
Ranks

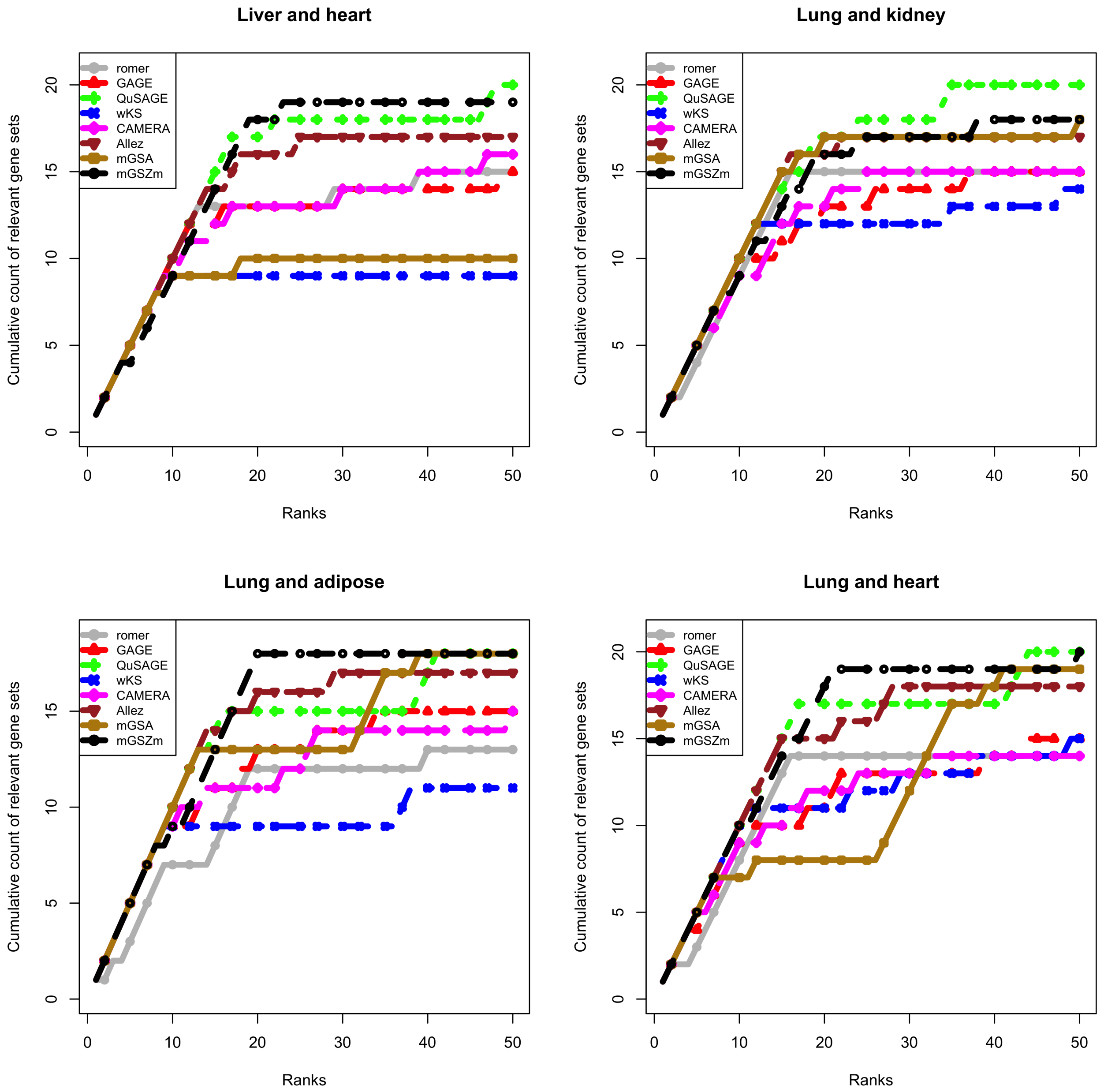
Liver and heart
Lung and kidney
Lung and adipose
Lung and heart
romer
GAGE
QuSAGE
wKS
CAMERA
Allez
mGSA
mGSZm
Cumulative count of relevant gene sets
Ranks

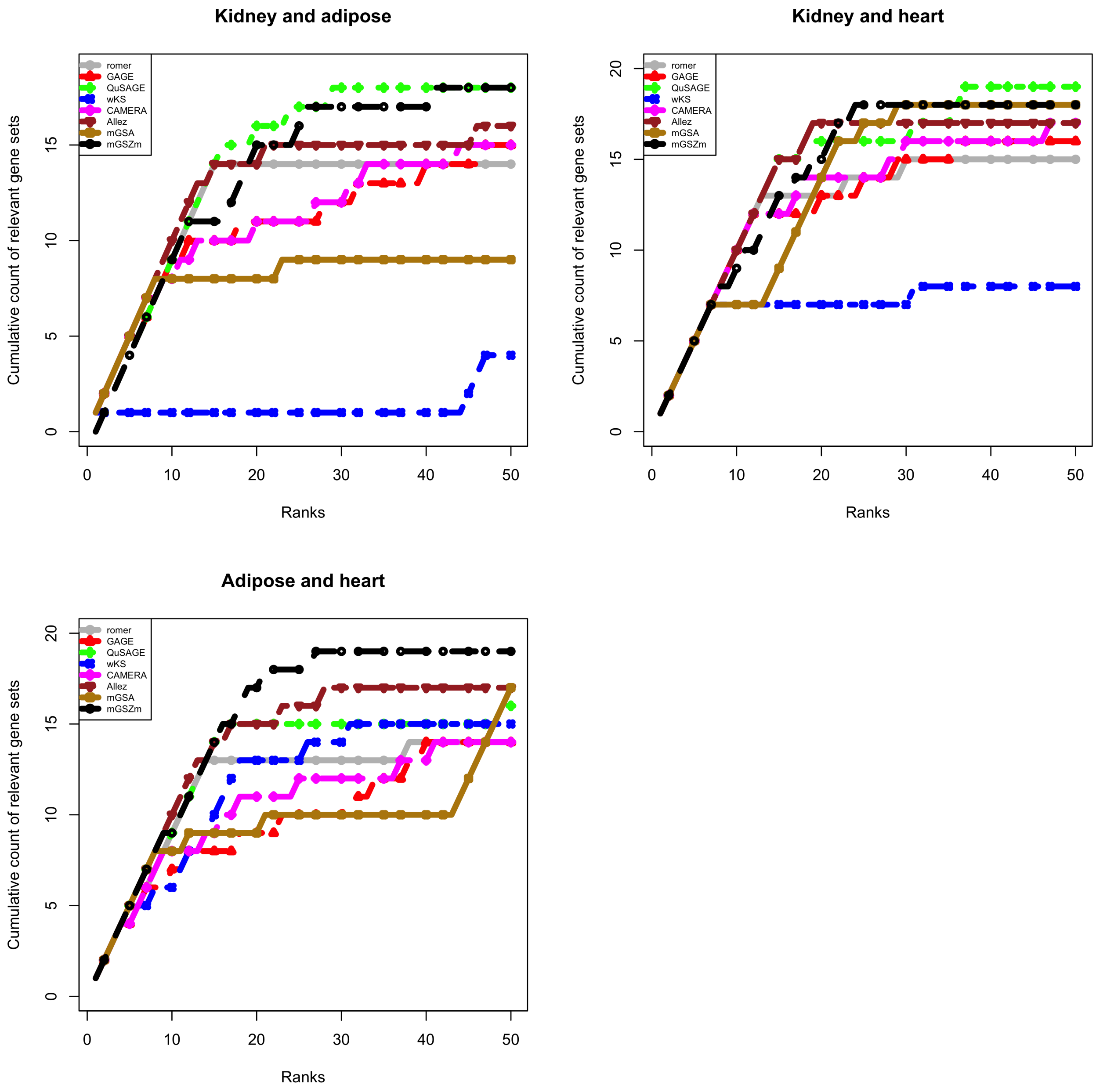

Figure 13: Tissue specific gene sets identified by eight compared gene set analysis methods from 15 pairwise comparisons of six different tissues in mouse tissue specific gene expression data. Figures represent cumulative count of tissue specific gene sets over the ranked list of top 50 gene sets reported by each of the compared methods.

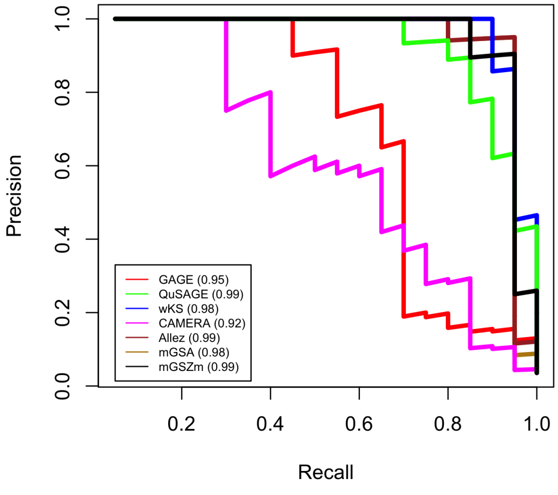

(a) Muscle and lung

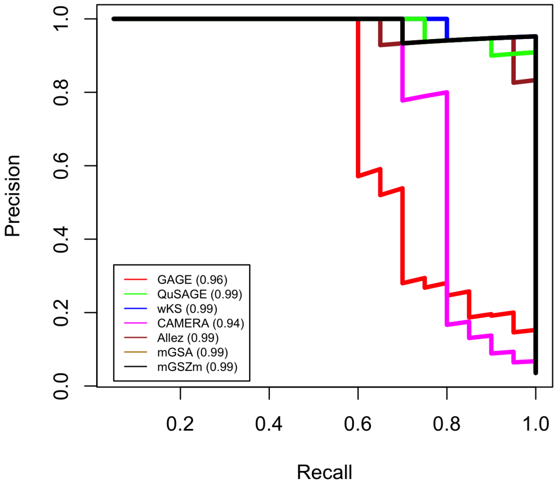

(b) Muscle and heart

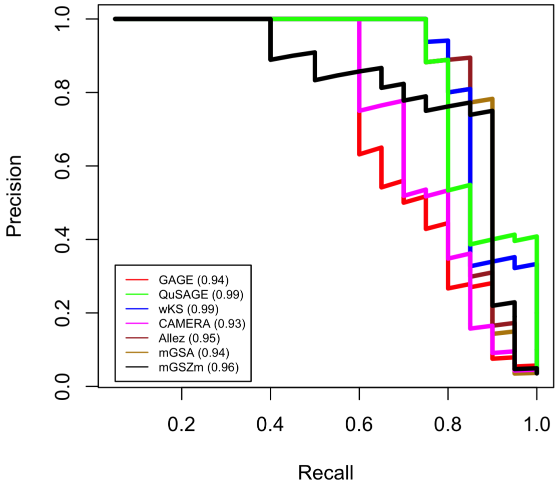

(c) Kidney and heart

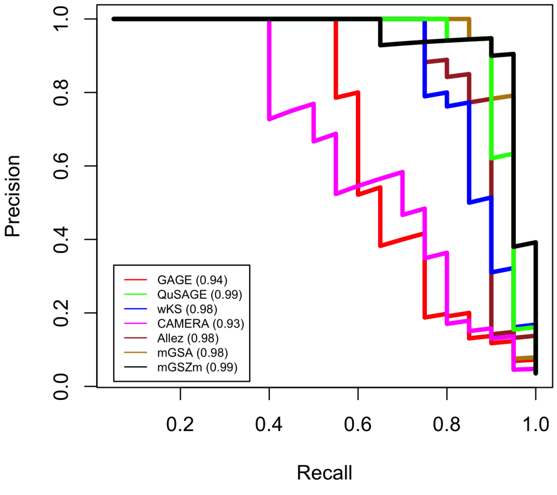

(d) Muscle and liver

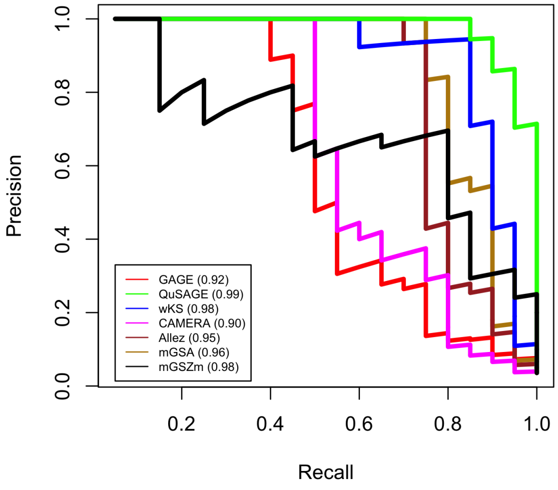

(c) Liver and adipose

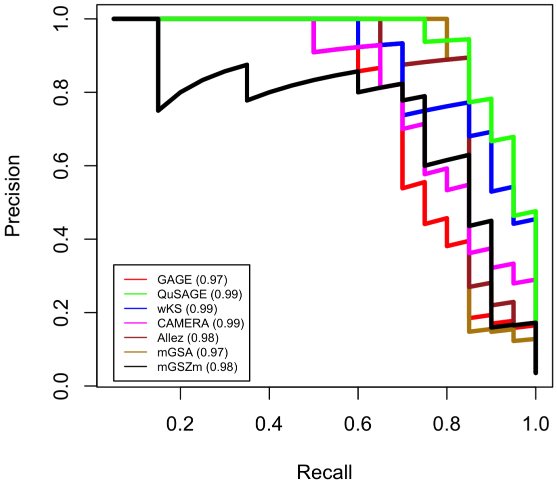

(d) Liver and kidney

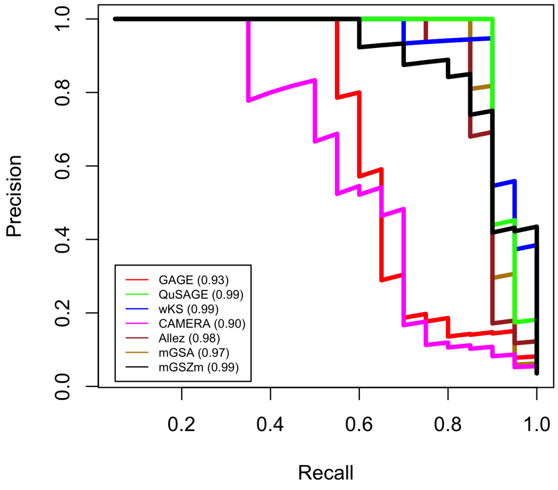

(c) Liver and lung

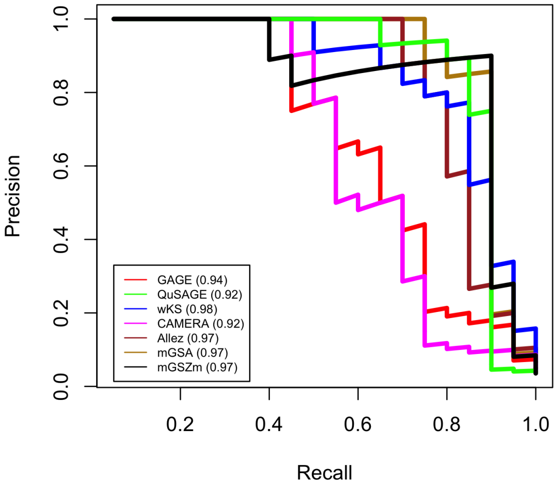

(d) Lung and adipose

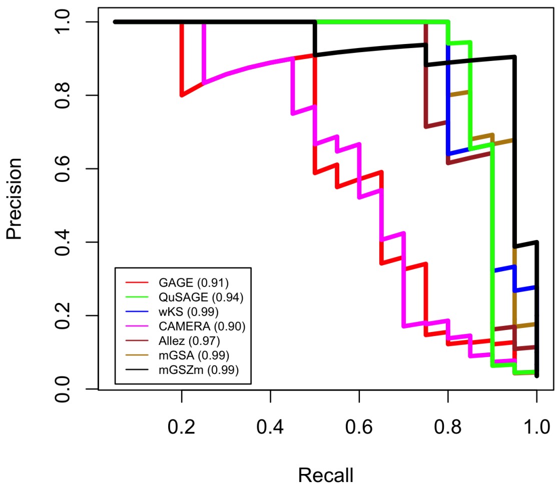

(c) Lung and heart

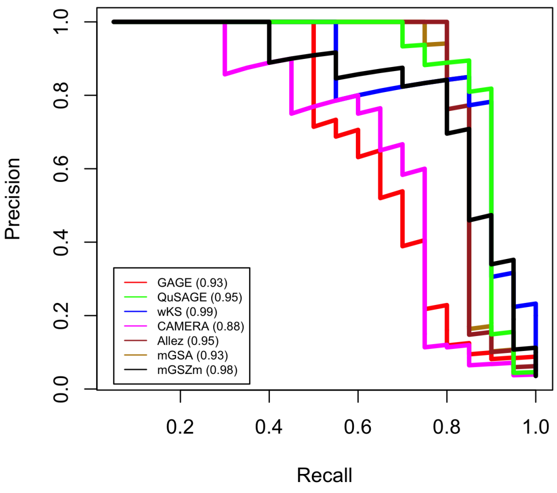

(d) Lung and kidney

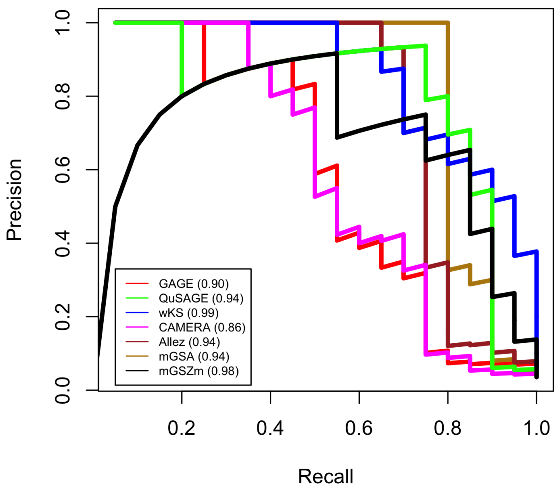

(c) Kidney and adipose

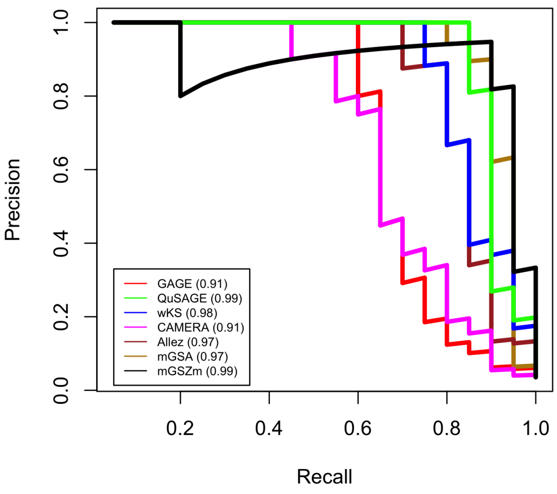

(d) Liver and heart

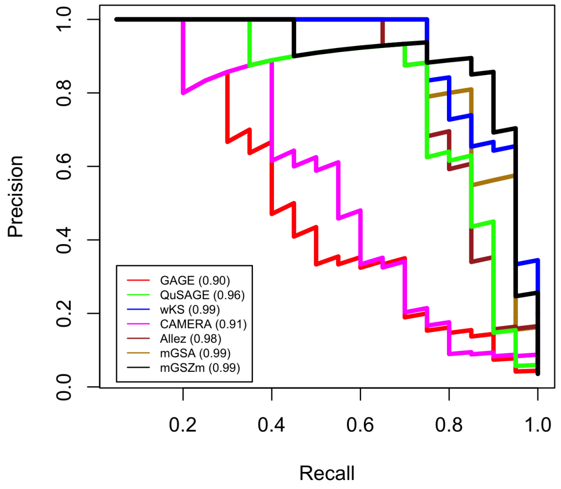

(e) Adipose and heart

Figure 14: Precision-recall plots for 15 pairwise comparisons of six different tissue samples in mouse tissue specific gene expression dataset.
